# A Comparative Analysis of Optimization Algorithms for Gastrointestinal Abnormalities Recognition and Classification Based on Ensemble XcepNet23 and ResNet18 Features

**DOI:** 10.3390/biomedicines11061723

**Published:** 2023-06-15

**Authors:** Javeria Naz, Muhammad Imran Sharif, Muhammad Irfan Sharif, Seifedine Kadry, Hafiz Tayyab Rauf, Adham E. Ragab

**Affiliations:** 1 Department of Computer Science, COMSATS University Islamabad, Wah Campus, Wah 47040, Pakistan; 2 Department of Computer Science, University of Education Lahore, Jauharabad Campus, Lahore 54770, Pakistan; 3 Department of Applied Data Science, Noroff University College, 4612 Kristiansand, Norway; 4 Department of Electrical and Computer Engineering, Lebanese American University, Byblos P.O. Box 13-5053, Lebanon; 5 Artificial Intelligence Research Center (AIRC), College of Engineering and Information Technology, Ajman University, Ajman 346, United Arab Emirates; 6 MEU Research Unit, Middle East University, Amman 11831, Jordan; 7 Centre for Smart Systems, AI and Cybersecurity, Staffordshire University, Stoke-on-Trent ST4 2DE, UK; 8 Industrial Engineering Department, College of Engineering, King Saud University, P.O. Box 800, Riyadh 11421, Saudi Arabia

**Keywords:** gastrointestinal tract disease, deep learning, features optimization, stomach diseases, features fusion, wireless capsule endoscopy, stomach cancer

## Abstract

Esophagitis, cancerous growths, bleeding, and ulcers are typical symptoms of gastrointestinal disorders, which account for a significant portion of human mortality. For both patients and doctors, traditional diagnostic methods can be exhausting. The major aim of this research is to propose a hybrid method that can accurately diagnose the gastrointestinal tract abnormalities and promote early treatment that will be helpful in reducing the death cases. The major phases of the proposed method are: Dataset Augmentation, Preprocessing, Features Engineering (Features Extraction, Fusion, Optimization), and Classification. Image enhancement is performed using hybrid contrast stretching algorithms. Deep Learning features are extracted through transfer learning from the ResNet18 model and the proposed XcepNet23 model. The obtained deep features are ensembled with the texture features. The ensemble feature vector is optimized using the Binary Dragonfly algorithm (BDA), Moth–Flame Optimization (MFO) algorithm, and Particle Swarm Optimization (PSO) algorithm. In this research, two datasets (Hybrid dataset and Kvasir-V1 dataset) consisting of five and eight classes, respectively, are utilized. Compared to the most recent methods, the accuracy achieved by the proposed method on both datasets was superior. The Q_SVM’s accuracies on the Hybrid dataset, which was 100%, and the Kvasir-V1 dataset, which was 99.24%, were both promising.

## 1. Introduction

Numerous researchers are recommending their methods for the precise identification and classification of gastrointestinal anomalies, skin lesions, and brain tumors. In the area of computer vision and medical image processing, this is a growing field of study. These research studies are usually based on the dataset obtained through imaging technologies such as computed tomography (CT), wireless capsule endoscopy (WCE), and magnetic resonance imaging (MRI). Gastric abnormalities incorporate esophagitis, bleeding, ulcer, and polyps. The most common gastrointestinal abnormalities that humans suffer from are bleeding, polyps, and ulcers [1]. These stomach abnormalities have turned into a main source of mortalities in people [2]. Around the world, stomach disease is the third-most significant reason for death among all malignant deaths [3]. Due to the recent environmental changes and ecological contamination in human dietary propensities, gastrointestinal system sicknesses have become progressively serious dangers to human wellbeing. The detection of Gastrointestinal Tract (GIT) tumors at the early stages will prevent many other GIT diseases from being more severe [4]. Since the last two decades’ rapid growth of technology, the utilization of machine learning, computer vision, and deep learning algorithms resulted in the extraordinary performance of several domains such as plants disease detection, surveillance, medical imaging for infections, and severe disease detection such as tumors [5]. Esophageal and gastric cancers are both common and deadly. Patients present most often after disease progression, and survival is therefore poor. Due to demographic variability and recent changes in disease incidence, much emphasis has been placed on studying risk factors for both esophageal and gastric cancers. Esophageal cancer (EsC), including squamous cell carcinoma (SCC) and adenocarcinoma, is considered as a serious malignancy with respect to prognosis and a fatal outcome in the great majority of cases. Esophageal carcinoma affects more than 450,000 people worldwide, and the incidence is rapidly increasing [6,7]. Gastric cancer accounts for 783,000 deaths each year, making it the third-most deadly cancer among males worldwide. In total, 8.3% of all cancer deaths are attributable to gastric cancer. The cumulative risk of death from gastric cancer, from birth to age 74, is 1.36% for males and 0.57% for females. Esophageal cancer can cause bleeding, and according to a publication in the American Journal of Gastroenterology, individuals with esophagitis have a significantly increased risk of developing esophageal cancer [8].

WCE is a noninvasive diagnostic method used for the careful examination of the bowel [9]. It is a painless examination as compared with existing traditional techniques [10,11]. WCE images play a very decisive role in the evaluation of stomach bleeding such as infections in the gastric tract (GI) and ulcers. A well-known technique, video capsule endoscopy (VCE), is also used for it [11,12]. When WCE is passed down the throat, it captures colored images for a medium time of 8 h that are received by a data recording device. Then, expert physicians analyze these images and make decisions about the disease [13]. It is a fact that standard endoscopic techniques diagnose the disease correctly, but they have the main disadvantage of causing discomfort and pain. To resolve these issues, the WCE modality is used. Many researchers have utilized WCE images for the evaluation of their proposed methods. Image classification is the most challenging task, especially in medical imaging. Doctors must spend a lot of time and effort categorizing and identifying ulcers, but doing so is crucial. To solve this problem, however, computerized methods have been created by many researchers. These techniques mainly rely on (a) familiarity with diverse ulcers, bleeding, and polyp images, (b) features such as color and texture, and (c) accuracy and execution time calculation. Researchers have recently developed a number of methods for automatically identifying diseased regions, especially focused on the color, texture, shape, and deep learning features. Due to difficulties including low-quality dataset pictures, color similarity between infected and healthy parts, differences in the texture and form of the infected area, and the necessity for feature engineering, detecting and classifying gastrointestinal disorders is a challenging endeavor [14].

The primary aim of this research is to propose a method that will recognize gastrointestinal abnormalities accurately with reduced computational time and improved results in terms of accuracy. The proposed method is based on the two deep learning models. There exist several challenges in this domain, such as images with low quality, noisy images, the selection of the appropriate method for quality enhancement, and suitable features extraction. Improving the quality of the dataset such that regions of interest become more highlighted and subtle information becomes prominent, strong feature selection, variation in the scalability of input data, and variation in texture information are also challenges. A gastrointestinal abnormality recognition and classification strategy is presented to tackle these limitations. The main steps of our proposed method are: (a) image pre-processing; (b) features engineering; (c) classification. The major contributions are:

▪ Contrast enhancement is one of the main contributions of this research work. In the pre-processing step, hybrid contrast enhancement methods are used to improve lesion contrast. The major steps performed in the pre-processing phase are 3D-box filtering, 3D-median filtering, HSI color transformation, and channels extraction. Contrast Limited Adaptive Histogram Equalization (CLAHE) is employed on the extracted channels and gives the output H_CLAHE, S-CLAHE, and I_CLAHE; in the next step, the saturation Weight-Map is applied on the S_CLAHE. In the last step of the pre-processing phase, un-sharp masking is employed on the concatenated output.
▪ A CNN model is designed from scratch with 23 layers, named XcepNet23. The proposed CNN model is pretrained on the CIPHER-100 dataset. Features engineering is performed on the extracted deep features.
▪ A hybrid features engineering methodology is proposed. Features vectors are obtained using the fine-tuned ResNet18 model, the proposed XcepNet23, and LBP features. The strong feature vector is created by combining the texture features and the extracted features from the two CNN models. The BDA, MFO, and PSO algorithms are used to optimize the fused feature vector.

The main objective is to develop a reliable and efficient model that can correctly identify and categorize digestive diseases. In comparison to current techniques, the research intends to obtain improved diagnostic accuracy, facilitating early identification and efficient treatment. The objective is to identify and categorize a wide range of digestive diseases by utilizing the strength of CNN and ML algorithms. This seeks to give professionals a thorough diagnostic tool for precisely identifying diseases. The rest of the paper is arranged as follows: The literature review is in Section 2, the concise narrative of the proposed methodology is in Section 3, and the quantitative experimental results are discussed in Section 4. Section 6 presents the paper’s conclusion.

## 2. Related Work

The automated detection of disease is an active area of research. Researchers have introduced numerous automated computer-aided diagnosis methods to help physicians. Most of the methods are suggested for malignant disease detection, including brain tumor segmentation and classification [15,16], glaucoma detection [17], lung cancer detection [18], and so on. The accurate detection of the infected region, such as ulcers and bleeding, is a most challenging task. There exist different methods that the researchers use for image enhancement based on gamma correction [19], image colorization [20], a geometric filter [21], the OTSU Threshold [22] and discrete Fourier-transform. A large memory and normally more than 8 h are required for the WCE examination. A real-time analysis algorithm is required to correctly analyze abnormal tissues or infected regions.

An algorithm is developed for automatic bleeding region detection [23] that mainly focuses on bleeding spot detection. It consists of the statistical analysis and shape analysis of the region of interest (ROI). This algorithm was tested on 30 different cases of capsule endoscopy and achieved 97% and 99% accuracy of specificity and sensitivity, respectively. Yuan et al. [24] came up with an approach for the detection of ulcers, polyps, and bleeding using the K-means clustering algorithm. They utilized WCE images and achieved an 88.61% average performance. The authors presented a procedure for bleeding region recognition from WCE imageries. In this method, pixels are assembled robustly based on the intensity and area through superpixel division instead of processing each pixel separately or through a uniform division of pixels [25]. A novel method is used in [26] for distinguishing healthy and bleeding regions. Bleeding is a major symptom of a disease. Different color models are used for the detection of bleeding, and experiments are performed by using local binary patter (LBP). Experiments show that by using CIE XYZ, an accuracy of 96.38% is achieved for the KNN classifier. The identification of problematic images has been an obstacle for specialists to deal with. An integrated saliency measure and the Bag of Features method are suggested to tackle this problem [27]. The proposed technique provides good classification and characteristics of polyps from WCE images. In [28], diverse measurable parameters are thought to accurately detect bleeding images such as the maxima, mean, minima, median, mode, variance, kurtosis, median, and skewness. In [29], images of ulcers and healthy people are categorized using the extraction of texture feature extraction. The contour let transform and log Gabor filter are used for texture features extraction. SVM receives the extracted texture features, which have the highest accuracy of 94.16%. Yuan et al. [30] suggested a saliency-based practice for the detection of ulcers. The proposed method comprises two main stages. Sample images of the ulcer are selected in the first step, whereas in step two, the multilevel super pixel method is used to segment infected regions that are joined in a single map. In [31], the authors proposed an approach for separating healthy and unhealthy pixels through different color spaces. RGB, HSV, YCbCr, etc. shading spaces are utilized for the extraction of texture features. Reduced FV is supplied to the SVM classifier for the classification of reduced features. They have shown a 97.89% classification accuracy. Reed T. Sutton et al. [32] carried out research in which they compared the performance of several deep learning algorithms. The authors utilized the HyperKvasir dataset and obtained 8000 images from the dataset. From the comparison of the CNN model, it is concluded that the DenseNet121 model achieved a maximum accuracy of 87.50%. A method for identifying stomach diseases was proposed by Nayar et al. [33]. The authors combined the CNN features with the improved Genetic Algorithm (GA). The feature extraction in this study is carried out with AlexNet. The computational time required for this method was 211.90 s, and the accuracy was 99.8%. Guanghua Zhang et al. [34] proposed a method for digestive tract tumor detection. In our previous study [35], a hybrid method was proposed for the classification of gastrointestinal diseases. The major steps involved in this methodology are pre-processing, texture features extraction, CNN features extraction, feature fusion, and classification. The highest classification accuracy achieved on the KVASIR dataset was 99.3%. The literature review depicts the pre-processing and features extraction steps used by most researchers for disease classification and detection. Moreover, some researchers have utilized a combination of different types of features for better classification results. As the prediction of ulcers, health, and bleeding from WCE images is greatly influenced by features that are to be extracted, therefore, in this research, our focus will be on the pre-processing step and features engineering phase.

A crucial phase that has a significant impact on prediction performance is improving the quality of the dataset. A hybrid strategy for enhancing the image quality is proposed in this study. The feature’s engineering phase is likewise basic in our proposed technique, wherein two kinds of features are extracted. These features are obtained utilizing CNN models and the Local Binary Pattern (LBP) algorithm. The CNN features are extracted using the proposed CNN model (XcepNet23) and the ResNet18 model. In the final phase, machine learning algorithms are utilized for classification. The following section provides a comprehensive explanation of the proposed method.

## 3. Proposed Methodology

**Figure 1:**
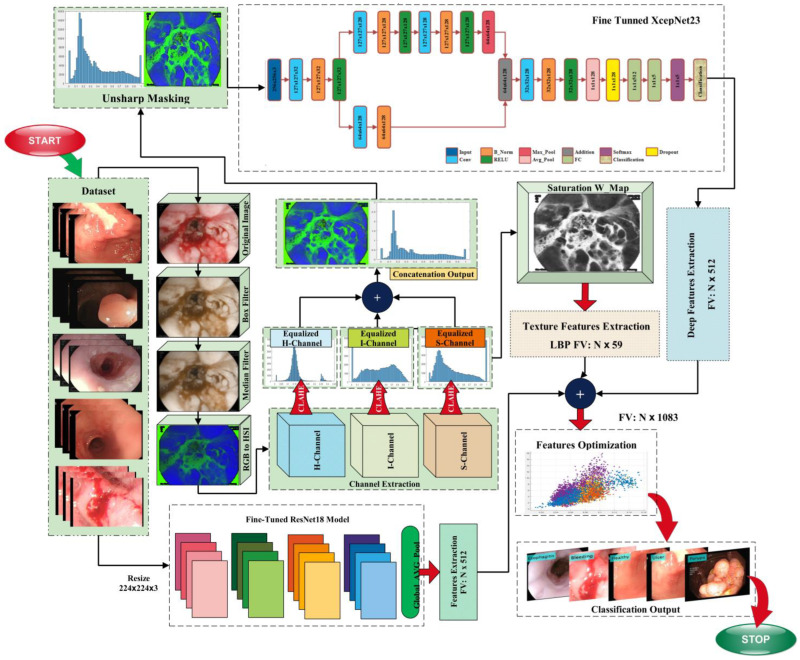
Proposed model block diagram.

The automatic detection of gastrointestinal diseases is a trending area of research in computer vision and image processing. Due to the identical color of cells in normal and infected WCE images, the classification of images is also an important task that is highly sensitive. The proposed method’s in-depth description is included in this section. This research proposes a method consisting of hybrid image processing, machine learning, and deep learning algorithms for the recognition of gastrointestinal abnormalities. This work is primarily based on five main phases, which are: (a) image augmentation; (b) hybrid pre-processing technique; (c) features extraction; (d) feature fusion and optimization; and (e) classification. The following sections provide a comprehensive explanation of these phases. The model that represents the proposed method is given in Figure 1.

### 3.1. Preprocessing

Preprocessing is the first phase of our method; the contrast of the image is improved to enhance the chromatic quality of the infected region. The pre-processing step has a great impact on different domains such as computer vision, medical imaging, biometrics, and surveillance. This step is performed in medical imaging due to the presence of numerous challenges such as the low contrast of the images, the presence of blur, noise artifacts, variations in illumination, color variations, and lightning effects. Image enhancement is performed to extract the most salient features and obtain accurate classification results. In this phase, different methods are utilized for contrast enhancement. The pre-processing step is further subdivided into major sub-steps, which are: data augmentation, 3D-box filtering, 3D-median filtering, RGB to HSI color transformation, channels extraction (H, S, I), CLAHE is employed on the extracted channels, which results in H_CLAHE, S_CLAHE, and I_CLAHE, a saturation weight map is employed on the S_CLAHE, and feature engineering is employed on the ensemble feature vector.

#### 3.1.1. Data Augmentation

**Figure 2:**
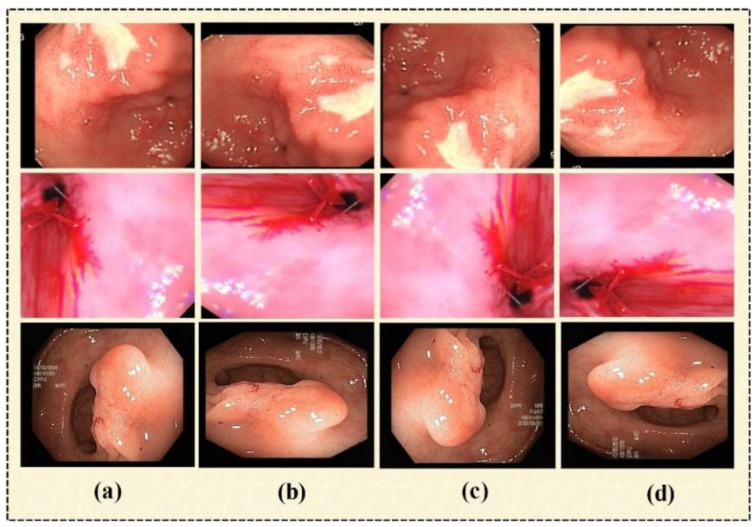
Sample images. (**a**) Original image, (**b**) 90°—right, (**c**) 180°—right, (**d**) 90°—left.

The availability of large datasets is still a challenge for researchers. For the training phase, deep learning models require a sizable quantity of data. Overfitting is a well-known issue that affects all researchers and is brought on using insufficient or tiny datasets. In this study, we used the data augmentation technique to expand the dataset size and balance the dataset. The research reveals that there are several image augmentation techniques, including image-flipping, mirrored images, and image rotation. In this paper, image rotation is utilized for the lossless augmentation of the dataset. We have rotated the original image in three angles, which are: 90°—right, 180°—right, and 270°—right (or 90°—left). Some sample output images of data augmentation are given in Figure 2.

#### 3.1.2. D-Box Filtering

**Figure 3:**
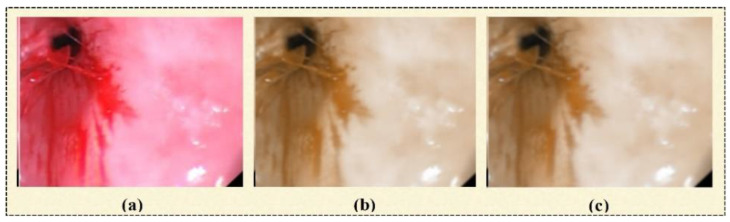
(**a**) Original image, (**b**) box-filter output image, (**c**) median-filter output image.

In this step of image preprocessing, a 3D-box filter [12] is employed for the initial image, as shown in Figure 3a. The output image of this step is shown in Figure 3b. It is used to smooth the image based on assigning an equal mask to the neighborhood pixels. It has three steps. In the first step, an RGB image I(s,d) is converted into three channels. A box filter (1’s pixel) is generated, which a size of 3×3. A mathematical representation of the box filter is given in Equation (1).

(1) $$Mask\left(h,v\right)=\sum_{j=1}^{3}\sum_{k=1}^{3}\left[Hj\right]\left[Vk\right]$$

where j Є H, k Є V, and h and v represent the rows and columns of the produced mask filter. Here, the mask size is 3×3=9, which is separately applied on all channels, as depicted in Equations (2)–(4).

(2) $$R\_mask(s,d)=Mask(h,v)[R\left(s,d\right)]$$

(3) G_mask(s,d)=Mask(h,v)[G(s,d)]

(4) B_mask(s,d)=Mask(h,v)[B(s,d)]

where $R\left(s,d\right)$ is the red channel and G(s,d) and B(s,d) represent the green and blue channels, respectively. R_mask(s,d) represents the kernel filter for the red channel, G_mask(s,d) is the kernel filter for the red channel, and B_mask(s,d) is the kernel filter for the blue channel. Equations (5)–(7) for channel extraction are as follows.

(5) $$R\left(s,d\right)=\frac{red}{I(s,d)}$$

(6) $$G(s,d)=\frac{green}{I(s,d)}$$

(7) $$B(s,d)=\frac{blue}{I(s,d)}$$

Updated pixel values and kernel filters are utilized to perform convolution operations. A new matrix is produced by using a null matrix with a size of 256 × 256 and the above-generated values of h = 3, v = 3, h_1_ = 256, and v_1_ = 256. The resultant new matrix is shown below in Equation (8).

(8) $$F_{c}\left(s,d\right)=\sum_{j=1}^{h_{M}}\sum_{k=1}^{v_{N}}\left[Z_{j,k}\left(s,d\right)+Mask\left(h,v\right)\right]$$

where j Є h_u_, k Є h_c_, h_u_ = 3, v_u_ = 3, h_M_ = h_u_ + h_1_ − 1, and v_N_ = v_u_ + v_1_ − 1. The symbols h_u_ and v_u_ represent updated values of rows and columns iterated up to 256 times. 2D images are produced by using F_c_(s, d) for each channel in the last step. Concatenating them results in a new, improved picture that amplifies the contrast between the diseased area and the surrounding area, as explained in Equations (9) and (10), respectively.

(9) $$B\_F\left(s,d\right)=\sum_{j=1}^{M}\sum_{k=1}^{N}I_{jxk}$$

(10) $$F_{n}\left(s,d\right)=\sum_{j}B\_Fj(sd)$$

where F*_n_*(s,d) represents the result of the 3D-box filter, as given in Figure 3b.

#### 3.1.3. D-Median Filtering

It is applied to the output image produced by the 3D-box filter. Noise issues are resolved through this filter [36]. The resultant image is shown in Figure 3c.

#### 3.1.4. HSI Color Transformation

The additive system, which can be obtained by combining all three channels—red, green, and blue—is the foundation of the RGB color model. The numbers that represent these colors are expressed as the set of three numeric values representing the intensity of three channels ranging from 0 to 255. The lowest value represents the black color (0, 0, 0), while the highest value is the representation of the white color (255, 255, 255). Another color model based on three parameters is the HSI model. In HSI color transformation, colors are encoded according to Hue, Saturation, and Intensity. Along with the RGB color model, this color system is used as an alternative to RGB color space in some color monitors [37]. RGB to HSI color transformation is applied to the outcome from the previous step. In this color wheel, the hue of the HIS transformation of intensity or color is represented by the angle measure. The second channel of the HSI color model is saturation. A color’s intensity or purity, especially in relation to its brightness, is referred to as saturation. It shows how much a color has been diluted with white or grey. The highly saturated color seems to be pure and vivid whereas, the de-saturated image is washed out. Low saturation indicates that there is dilution with white light, whereas high saturation means the color is pure and free from dilution. The third component is the intensity (brightness), which depends on the saturation and hue component. The mathematical equations for the conversion of RGB to the HSI color space [38] are given in Equations (11)–(14):

(11) I=(r+g+l)/3

(12) $$H=\cos^{-1}\left(\frac{0.5[((r-g)+(r-l)}{\sqrt{{\left(r-g\right)}^{2}+\left(r-l\right)\left(g-l\right)}}\right)$$

(13) $$if\;l>g\;then\;H=360^{{}^{\circ}}-H$$

(14) $$S=1-(\frac{3}{r+g+l}){\left[\min\left(r,g,l\right)\right]}^{}$$

#### 3.1.5. Contrast Limited Adaptive Histogram Equalization

Numerous researchers in image processing have used CLAHE to improve contrast by calculating distinct histograms for each image region; this algorithm improves contrast. These histograms are utilized in the next steps for the equal distribution of gray levels across the range of images. After the color transformation, in the next step, the H, S, and I channels are extracted, and CLAHE [39] is applied to all three extracted channels. After CLAHE, three output images are obtained, which are H_CLAHE, S_CLAHE, and I_CLAHE. The ensemble feature vector ($\phi_{Ensemble}^{CLAHE}$) is obtained by concatenating the H_CLAHE, S_CLAHE, and I_CLAHE. After the CLAHE step, the Chromatic weight map (C_W_MAP) is employed on the S_CLAHE, and an improved image is obtained (${S\_CLAHE}_{Improved}$) as an output of this step. The other two CLAHE outputs remain unchanged. The ${S\_CLAHE}_{Improved}$ is further utilized for the texture features extraction. The $\phi_{Ensemble}^{CLAHE}$ is sharpened to improve the quality. In the next phase, the $\phi_{Ensemble}^{SHARP}$ image is obtained, which is further utilized for the proposed CNN model for the deep features extraction.

### 3.2. Feature Extraction

**Figure 4:**
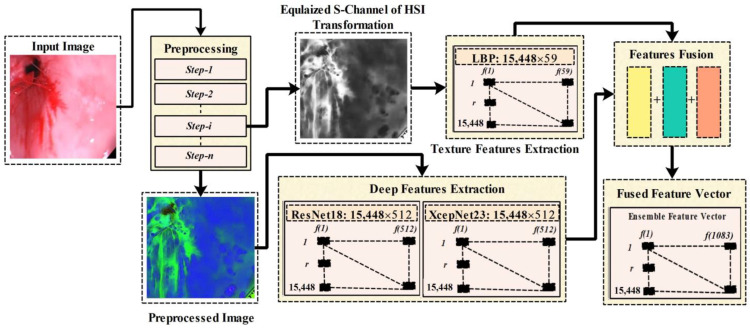
Proposed features engineering process.

Feature Extraction and optimal feature selection are significant phases that are necessary to achieve the accurate detection and classification of GIT abnormalities. In this research, we have utilized the Local Binary Pattern for the texture features extraction, and two CNN model are utilized for the deep features extraction. In Figure 4, there is an overview of the phases from preprocessing features extraction, deep features extraction, and features fusion. Whereas in the preprocessing phase we have performed several steps such as data augmentation, box-filtering, median filtering, and RGB-to-HSI conversion, in the preprocessing block of Figure 4, step-i represents the extracted Equalized S-channel output of the preprocessing phase, and Step-n represents the final output image of the preprocessing phase. The equalized S-channel image is utilized for the LBP features extraction that is later ensembled with the extracted deep learning features. A block diagram representing the proposed features engineering process is given in Figure 4.

#### 3.2.1. Local Binary Pattern

LBP [40] is an important method utilized by many researchers for the extraction of texture information. It takes a grayscale image as the input after that variance and mean are calculated for all of the intensities (pixels) of the image. In this research project, we have utilized the S_CLAHE for the LBP features extraction. This algorithm returned the feature vector of dimension N×59. The mathematical formulation of LBP is given below in Equations (15) and (16):

(15) $$LBP\left(Q,R\right)=\sum_{Q=0}^{Q-1}Y(E_{Q}-E_{C})2Q$$

(16) $$Y\left(x\right)=\left\{\begin{array}{c} 1\;\;\;\;\;\;\;ifQ\geq 0\\ 0\;\;\;\;\;otherwise \end{array}\right.$$

In this formulation, the neighborhood intensities are given by Q, the radius is given by R, $E_{C}$ represents the current pixel intensity, and $E_{Q}$ represents the neighboring pixel intensity. Y(x) refers to the thresholding function.

#### 3.2.2. Deep Learning Features

Inspired by the usefulness of deep learning in the surveillance and agriculture domain, several researchers have employed deep learning models for disease recognition. Deep learning models have resulted in a great breakthrough in terms of disease detection and classification performance. A CNN model consists of multiple layers comprising the input, convolution, batch normalization, RELU, pooling, softmax, and classification layers. Each layer is responsible for performing a specific task. A detailed description of the CNN models utilized in this research is given in the following section:

##### ResNet18

**Figure 5:**
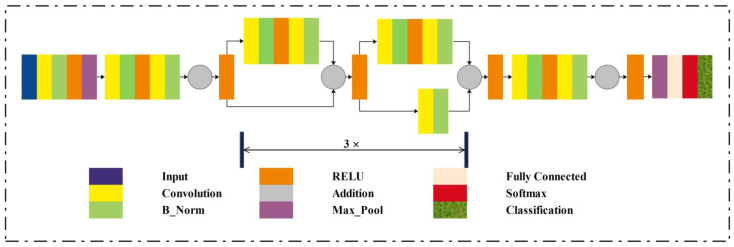
Layered architecture of the ResNet18 model.

There are different versions of Residual Network (ResNetXX) architecture [41], where “XX” specifies the total number of layers. For the deep feature extraction in this study, the ResNet18 version was used. There are 72 layers in this architecture, with 18 of them representing the model’s 18 deep layers. The ResNet18 model is originally pre-trained on the thousand categories of the ImageNet dataset, consisting of the ResNet18 model containing 11,511,784 trainable parameters. The primary aim of this model is enabling a large number of convolution layers. The network performance becomes saturated or even degraded due to the presence of a vanishing issue. A vanishing gradient arises when there are multiple layers, and the continuous multiplication results in an even smaller gradient than the previous one; this situation leads to the network performance degradation. In the ResNet model, a new idea is presented to tackle the vanishing gradient problem, which is “skip connection”. The skip connections resolved the vanishing gradient problem by again utilizing the previous layer activations. Skip connections compress the network, and the network starts learning faster than before. For the training, a ratio of 70:30 is utilized for the training and testing. We have utilized five-fold cross-validation. Figure 5 gives the visual representation of the layered ResNet18 model architecture.

##### Proposed XcepNet23

**Figure 6:**
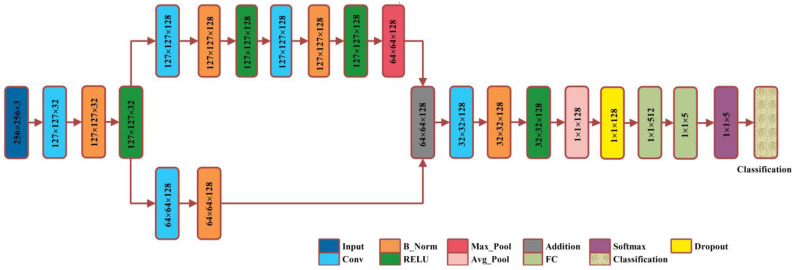
Proposed XcepNet23 layered architecture.

A smart deep learning model with 23 layers is proposed for this research work, named XcepNet23. The layered architecture of the proposed model follows the architecture of the original Xception network model that has a depth of 71 layers. The XcepNet23 model is designed from scratch and pre-trained on the CIFER-100 dataset. After the pre-training step, we trained it on our enhanced dataset. We have employed five-fold cross-validation, splitting the dataset into the ratio of 70:30 for training and testing, respectively. The parameters settings utilized for training are: the sgdm optimizer, an initial learning rate of 0.01, 30 max epochs, a mini_Batch_size of 64, and shuffling on each epoch is performed. The layered architecture of XcepNet23 is given in Figure 6.

#### 3.2.3. Features Optimization

In the literature, we have observed that authors have utilized feature fusion to obtain a strong resultant set of features. There may be some redundant features that reduce the performance of the method. In the last decade, methods based on metaheuristic algorithms have been viewed as the most proficient and solid improvement strategies. These algorithms have been broadly utilized for the improvement of the performance of real-world issues. In this research, we have utilized feature optimization algorithms to extract the optimal features among the total extracted features. Optimization algorithms help to get rid of redundant and non-optimal features. Many algorithms can be used for selecting the best features. It is observed in the literature that authors have utilized BDA [2], MFO in combination with the Crow Search algorithm (CSA) [42], PSO and CSA [43], Enhanced Crow Search and Differential Evaluation, and Grasshopper [44]. Inspired by these studies, in this paper, we used three algorithms inspired by nature to optimize features, which are BDA, MFO, and PSO. The following sections briefly overview the various optimization techniques under consideration. We have one feature vector after the features fusion step denoted by $\phi^{Fused}$, having the dimension N×1083. Optimization algorithms are applied on the $\phi^{Fused}$ to obtain the optimal set of features. The obtained feature vector is optimized using BDA, MFO, and PSO. The mathematical formulation of optimization algorithms is as follow:

The BDA [45] mimics the swarming dragonflies (DF) pattern. The fact-finding and shady systems of DA [46] are demonstrated by the association of dragonflies in keeping away from the foe and finding the source of food. The dragonfly algorithm mathematical formulation is given in the following Equations (17)–(25). Separation is formulated as:

(17) $$S_{i}=-\sum_{i=1}^{N}X-X_{i}$$

where X specifies a DF position in a space that is M-dimensional. *N* and $X_{i}$ represent the number of neighbor individuals and the position of the neighbor individual, respectively.

Alignment permits speed matching of the individual in a sub-swarm/swarm. The mathematical formulation of Alignment is as follows:

(18) $$A_{i}=\frac{\sum_{i=1}^{N}V_{i}}{N}$$

where *N* represents the neighbor individuals and $V_{i}$ represents the neighbor’s individual velocity. Cohesion alludes to the deviation of the ongoing individual toward the focal point of the mass of the neighbor individual.

(19) $$C_{i}=\frac{\sum_{i=1}^{N}X_{i}}{N}-X$$

where $X_{i}$ specifies the individual neighbor position.

Attraction is also an important behavior; it specifies that the source of food should be the attraction for the individuals. Mathematically, the attraction behavior is formulated as:

(20) $$L_{P}=X_{P}-X$$

where $X_{P}$ indicates the source food’s position.

Distraction indicates the situation in which an individual should not get closer to the enemy. The individual should get away from predators. The mathematical formulation of distraction is as follows:

(21) $$E_{i}=X_{E}-X$$

where $X_{E}$ represents the location of the enemy.

These five behaviors control the DFs’ movement in DA. The step vector is calculated to update the location of each dragonfly:

(22) $$∆X_{i}(T+1)=(sS_{i}+\alpha A_{i}+cC_{i}+fL_{P}+eE_{i})+w∆X_{i}(T)$$

where T signifies the current iteration, s is the separation weight, α is the alignment weight, c is the weight of cohesion, f is the food weight, e is the weight of the predator, and w is the weight of inertia. In the initial algorithm for dragonflies, the locations of the DFs are restructured using the following equation:

(23) $$∆X_{i}\left(T+1\right)=X_{i}\left(T\right)+∆X_{i}\left(T+1\right)$$

The locations vectors of BDA are updated as follows:

(24) $$X_{i}^{k}\left(T+1\right)=\left\{\begin{array}{c} 1-X_{i}^{k}\left(T\right)\;\;\;\;\;\;Rand<Tf(∆X_{i}^{k}(T+1))\\ X_{i}^{k}\left(T\right)\;\;\;\;\;\;\;\;\;\;Rand\geq Tf(∆X_{i}^{k}(T+1)) \end{array}\right.$$

(25) $$Tf\left(∆X\right)=\left|\frac{∆X}{\sqrt{{∆X}^{2}+1}}\right|$$

where *Rand*, ∆X*,* $X_{i}^{k},\;and\;Tf$ represent the number that is randomly generated (range: 0–1), the step vector, the *k*th position of the *i*th dragonfly, and the transfer function, respectively. The ensemble feature vector $\phi^{Fused}$ is processed using the BDA algorithm and obtains the optimized feature vector with the dimensions N×384.

In this research, we have also utilized the Moth–Flame Optimization algorithm [47] for feature optimization. There are three major steps that the moths follow: population creation, position updating, and updating the final amount of the flame. Here, the Moths population and Fitness function value can be mathematically expressed as given in Equations (26)–(34):

(26) $$W=\left[\begin{array}{c} \begin{array}{ccc} w_{1,1} & w_{1,2} & \ldots \\ w_{2,1} & w_{1,2} & \ldots \\ \vdots & \vdots & \ddots \end{array}\\ \begin{array}{ccc} w_{z,1} & w_{z,2} & \ldots \end{array} \end{array}\begin{array}{c} w_{1,n}\\ \begin{array}{c} w_{1,n}\\ \vdots \end{array}\\ w_{z,n} \end{array}\right]$$

(27) $$OW=\left[\begin{array}{c} \begin{array}{c} {OW}_{1}\\ {OW}_{2}\\ \vdots \end{array}\\ {OW}_{z} \end{array}\right]$$

(28) $$F=\left[\begin{array}{c} \begin{array}{ccc} F_{1,1} & F_{1,2} & \ldots \\ F_{2,1} & F_{1,2} & \ldots \\ \vdots & \vdots & \ddots \end{array}\\ \begin{array}{ccc} F_{z,1} & F_{z,2} & \ldots \end{array} \end{array}\begin{array}{c} F_{1,n}\\ \begin{array}{c} F_{1,n}\\ \vdots \end{array}\\ F_{z,n} \end{array}\right]$$

(29) $$OF=\left[\begin{array}{c} \begin{array}{c} {OF}_{1}\\ {OF}_{2}\\ \vdots \end{array}\\ {OF}_{z} \end{array}\right]$$

where the matrix *W* represents the moth’s population and the flame is represented by the F, *n*, and *z* denoting the number of dimensions and the number of moths. After that, the moths’ positions are altered to obtain the best global solutions. The following function is chosen for the optimization challenge’s best global solution:

(30) MFO=(P,Q,S)

where P is a function, P generates the fitness function corresponding to the randomly generated population $P:\emptyset \to \left\{W,OW\right\}$, and the Q function is the main function. Q is responsible for the moth movement around the search space Q:W→W, and the S function returns false or true based on the termination criteria S, which is formulated as $S:\left\{true,false\right\}.$ The mathematical formulation of the moth logarithmic spiral for the updating mechanism is given as follows:

(31) $$W_{i}=S(W_{i},F_{j})$$

(32) $$S\left(W_{i},F_{j}\right)=D_{i}\cdot e^{cr}\cdot \cos\left(2\pi r\right)+F_{j}$$

(33) $$D_{i}=\left|F_{j}-W_{i}\right|$$

(34) $$Selected_{Flame}=round\left(M-k*\frac{M-1}{T}\right)$$

where $D_{i}$ indicates the distance between the *j*th flame and the *i*th moth, c is a constant that defines the logarithmic spiral shape, and r is a random no. between the range −1 and 1. Equation (34) M denotes the max number of flames, the total number of iterations is denoted by T, and k represents the current iteration. $Selected_{Flame}$ is passed to the Equations (26)–(29), and the fitness function is calculated. The MFO technique that was mentioned earlier is used to process the fused feature vector $\phi^{Fused}$ and obtain the optimized feature vector with the dimensions N×437.

In this article, the third optimization algorithm that we have utilized is the PSO Algorithm [48]. PSO is a nature-inspired algorithm that is derived from the survival behavior of swarms. This algorithm’s primary goal is optimization or picking the best solution from the available possible solutions. For a K-Dimensional search space, where we have k particles, the vector for the *j*th particle of the swarm will be represented as $P_{j}={(p}_{j1},p_{j2},\ldots ,p_{jK})$, whereas, the prior optimal position of the *j*th particle is $l_{j}={(l}_{j1},l_{j2},\ldots ,l_{jK})$, which returns the optimal fitness value. Here, L symbolizes the particle’s lowest function value, and the velocity of the *j*th particle can be expressed as $V_{j}=({(v}_{j1},v_{j2},\ldots ,v_{jK})$. The following mathematical formulation represents the particles’ updating process:

(35) $$v_{id}^{y+1}=I\times v_{id}^{y}+c1\times r\left(\right)\times \left(l_{id}^{y}-p_{id}^{y}\right)+c2\times R\left(\right)\times (l_{gd}^{y}-p_{gd}^{y})$$

(36) $$p_{id}=p_{id}+v_{id}$$

where *c*1 and *c*2 denote the positive constant numbers of cognitive and social parameters, respectively. Here, *R*() and *r*() are the two functions that produce pseudorandom numbers within a certain range [0, 1]. This algorithm returned a feature vector of N×428 dimensions.

## 4. Experimental Results and Analysis

The dataset consists of thousands of images which are unhealthy and healthy. Unhealthy images are further categorized into two classes: ulcers and bleeding. In this work, several machine-learning algorithms are used to classify the data. To evaluate the findings presented in this paper, each of the six classifiers listed below is taken into consideration: Fine Tree (F_Tree) [49], Coarse Tree (C_Tree) [50], Quadratic SVM (Q_SVM) [51], Fine Gaussian SVM (F_G_SVM) [52], Coarse KNN (C_KNN) [53], and Ensemble Subspace Discriminant (E_S_Disc) [54]. The classifier models include Fine Tree (F_Tree) and Coarse Tree (C_Tree), which are decision tree-based models optimized for fine and coarse-grained classification tasks, respectively. Quadratic SVM (Q_SVM) is a support vector machine model that utilizes kernel functions to handle non-linearly separable data. Fine Gaussian SVM (F_G_SVM) employs a Gaussian kernel to model the probability density function of the input data. Coarse KNN (C_KNN) is a k-nearest neighbor model optimized for coarse-grained classification tasks. Ensemble Subspace Discriminant (E_S_Disc) is an ensemble model that combines multiple classifiers operating on different subspaces of high-dimensional data. All the experiments are performed on MATLAB 2020a, Core i5-7th Generation with 8 GB RAM.

### 4.1. Dataset

**Figure 7:**
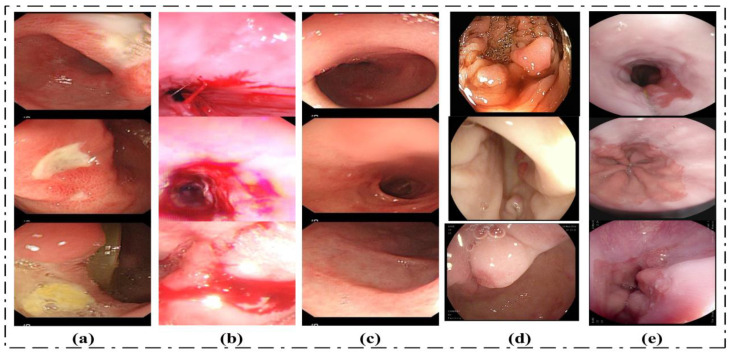
Sample dataset images: (**a**) Ulcer, (**b**) Bleeding, (**c**) Healthy, (**d**) Polyps, (**e**) Esophagitis.

Two datasets are utilized to evaluate the proposed method in this research paper. The considered datasets include: (1) the KVASIR_V1 Dataset and (2) the Hybrid_Dataset. The Hybrid_Dataset utilized in this research work consists of five classes: Bleeding, Ulcer, Esophagitis, Polyps, and Healthy. The dataset for this research is collected from different sources. Ulcer, Healthy, and Bleeding images are acquired from Amna Liaqat et al. [12] (each class contains 3000 images). The Esophagitis images are taken from the Kvasir dataset, [55]. Using the data augmentation technique, the volume of data for esophagitis images is increased without reducing the features in the original images. The fifth class of our dataset is polyps. Polyps’ images are collected from two datasets named the Kvasir and CVC datasets. The proposed method is evaluated using a total of 15,448 images. The second dataset considered for this research work is the KVASIR_V1 dataset. This dataset consists of eight classes, and the total number of images in this dataset is 4000 [55]. We have used all classes of this dataset. Some sample images are given in Figure 7.

### 4.2. Performance Measures

In this research, the execution of the suggested classification method is assessed on different performance procedures, which are the execution time, precision (PRE), accuracy (ACC), F1-Score (F1), specificity (SPE), sensitivity (SEN), Cohen’s Kappa score, and Matthews correlation coefficient (MCC). Mathematically, these performance measures are given from Equations (37)–(43). In this research, we have utilized five-fold cross-validation to assess all of the quantitative results.

(37) $$SEN=\frac{TPV}{TPV+FNV}$$

(38) $$SPE=\frac{TNV}{FPV+TNV}$$

(39) $$PRE=\frac{TPV}{TPV+FPV}$$

(40) $$F1=2*\left(\frac{SEN*PRE}{SEN+PRE}\right)$$

(41) $$ACC=\frac{TPV+TNV}{TPV+TNV+FPV+FNV}$$

(42) $$MCC=\frac{TPV\times TNV-FPV\times FNV}{\sqrt{\left(TPV+FPV)(TPV+FNV)(TNV+FPV)(TNV+FNV\right)}}$$

(43) $$Kappa\_Score=\frac{p_{o}-p_{e}}{1-p_{e}}$$

### 4.3. Results

Experiments are divided into five categories. The first category contains the results of the proposed methodology without optimizing the features, and the results will be taken on the fused extracted feature vector that is $\phi^{Fused}$. In the second, third, and fourth experiments, the fused feature vector $\phi^{Fused}$ is further optimized using three different algorithms. The optimized feature vector obtained as the output is fed to the machine learning classifiers. We have evaluated these experiments on two datasets (the description of datasets is given in the previous section).

#### 4.3.1. Experiment 1: Results on the $\phi^{Fused}$ Feature Vector

The $\phi^{Fused}$ feature vector is extracted by employing fusion on three different features that are extracted using: the ResNet_18 model, the proposed XcepNet23 model, and texture features extracted using the LBP method. We have performed this experiment on two datasets: the Hybrid_Dataset and the Kvasir_V1 dataset. After preprocessing the datasets, two types of CNN features and LBP features are extracted from the dataset. The obtained features of the Hybrid_Dataset returned a feature vector mathematically denoted as $\phi^{Fused}.$ The feature vectors obtained from the ResNet18 model and XcepNet23 are of N×512 dimensions, where N represents the Hybrid_Dataset’s total number of images. A feature vector of N×59 is obtained through the LBP. After the fusion of these three vectors, a fused feature vector is obtained, and the dimensions of $\phi^{Fused-1}$ are N×1083; these features are used by classifiers to identify and classify GIT abnormalities. Q_SVM has achieved 100% accuracy in 124.94 s. In terms of the other performance metrics, such as the SEN, SPE, PRE, and F1 score, Q_SVM performed well. E_S_Disc achieved the second-highest accuracy of 99.98% in 483.66 s, and F_Tree achieved the third-highest accuracy of 99.86% in 32.840 s. C_Tree had the worst classification performance, with an accuracy of 82.39%. Table 1 displays the quantitative findings of this experiment.

**Figure 8:**
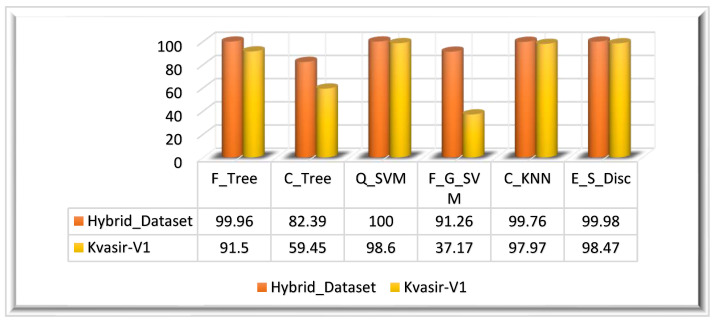
Experiment 1 results.

Similarly, preprocessed Kvasir-V1 images are utilized for feature extraction. The feature vectors obtained from the ResNet18 model and XcepNet23 are of M×512 dimensions, where M symbolizes the total number of images in the Hybrid_Dataset. A feature vector of M×59 is obtained through the LBP. In the next step, feature fusion is performed to obtain a strong set of features. The ensemble feature vector obtained from the Kvasir_V1 dataset is of dimensions M×1083. A fused feature vector is produced when these three extracted feature vectors are an ensemble that is denoted by $\phi^{Fused-2};$ the ensemble feature vector is utilized for the classification. The highest classification accuracy achieved on the Kvasir_V1 dataset is 98.60% on the Q_SVM classifier in 41.758 s. With 98.47%, E_S_Disc achieved the second-highest accuracy, while C_KNN came in third with a 97.97% accuracy. The results of this experiment are depicted graphically in Figure 8 for both datasets.

#### 4.3.2. Experiment 2: Results on the $\phi_{BDA}^{Fussed}$ Feature Vector

The ensemble feature vectors $\phi^{Fused-1}\;and\;\phi^{Fused-2}$ are optimized using the BDA algorithm. In this experiment, the results are taken on the $\phi_{BDA}^{Fussed}$ feature vector. For Hybrid_Dataset, the BDA algorithm returned a feature vector with the dimensions N×384. The optimized feature vector is utilized for the classification of the dataset. For the evaluation of BDA-optimized features, we have utilized multiple classifiers. From the results, it can be observed that Q_SVM has achieved 100% accuracy in 58.140 s. Q_SVM performed well compared to the other classifiers. E_S_Disc achieved the second-highest accuracy of 99.97% in 114.66 s, and C_KNN achieved the third-highest accuracy of 97.72% in 264.70 s. C_Tree had the worst classification performance, with an accuracy of 85.73%. Table 2 shows the results of this experiment.

**Figure 9:**
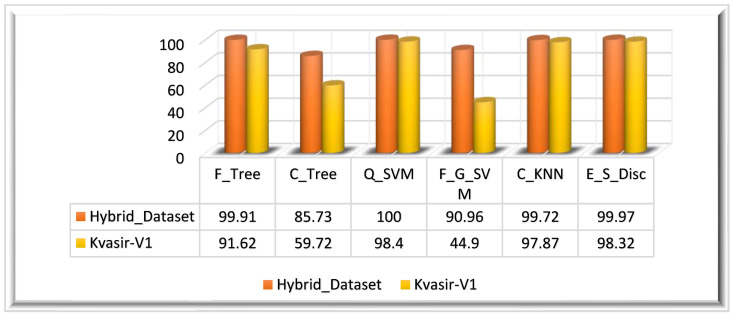
Experiment 2 results.

In this experiment, we have also computed the $\phi_{BDA}^{Fussed}$ feature vector on the Kvasir_V1 dataset. The ensemble feature vector obtained from the Kvasir_V1 dataset is of M×1083 dimensions. The ensemble feature vector is optimized using the BDA algorithm. The optimized feature vector has M×499 dimensions. The highest classification accuracy of this experiment (Kvasir_V1 dataset) is 98.40%, achieved on the Q_SVM classifier in 22.685 s. On the Kvasir_V1 dataset, the E_S_Disc classifier achieved the second-highest accuracy of 98.32% in 66.739 s, and C_KNN achieved the thirst-highest accuracy of 97.87%. F_G_SVM performed worst among all classifiers, achieving a 44.90% accuracy in 92.103 s. The visual representation of the results is given in Figure 9 for both datasets.

#### 4.3.3. Experiment 3: Results on the $\phi_{MFO}^{Fussed}$ Feature Vector

Experiment 3 is based on the $\phi_{MFO}^{Fussed}$ feature vector. The ensemble feature vectors $\phi^{Fused-1}\;and\;\phi^{Fused-2}$ are optimized using the MFO algorithm; this algorithm returned a feature vector with the dimensions N×437 for the Hybrid_Dataset. Table 3 presents the quantitative results of this experiment. For the evaluation of MFO-optimized features, we have utilized multiple classifiers. From the results, it can be observed that Q_SVM has achieved 100% accuracy in 67.941 s. E_S_Disc, one of the classifiers that was assessed, came in second, with an accuracy rating of 99.97%, trailing only F_Tree (99.76%). The least effective model, C_Tree, has a classification accuracy of 85.66%.

Experiment 3 is also evaluated on the Kvasir_V1 dataset. The MFO-optimized feature vector $\phi_{MFO}^{Fussed}$ is obtained by employing the MFO algorithm on the fused feature vector ($\phi^{Fused-2})$. The ensemble feature vector optimized using the MFO algorithm returned a feature vector of M×529 dimensions. The optimized feature vector $\phi_{MFO}^{Fussed}$ is classified using multiple classifiers. The highest classification accuracy of this experiment (Kvasir_V1 dataset) is 98.42%, achieved on the E_S_Disc classifier in 60.429 s.

**Figure 10:**
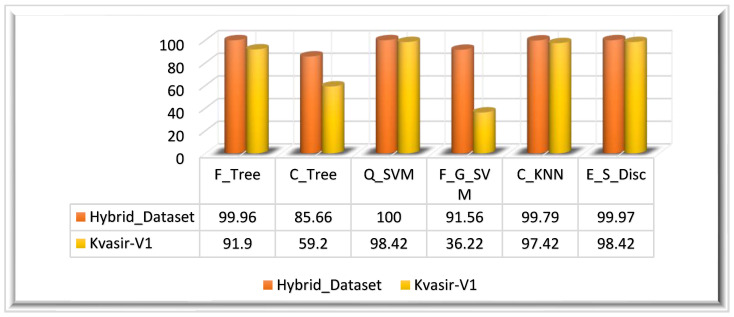
Experiment 3 results.

On the Kvasir_V1 dataset, the Q_SVM classifier achieved the second-highest accuracy of 98.37% in 36.044 s, and C_KNN achieved the thirst highest accuracy of 97.42%. F_G_SVM performed worst among all classifiers by achieving a 36.22% accuracy in 102.24 s. The graphical representation of the results is given in Figure 10 for both datasets.

#### 4.3.4. Experiment 4: Results on the $\phi_{PSO}^{Fussed}$ Feature Vector

In Experiment 4, we utilized the PSO algorithm for feature optimization; it is primarily reliant on the $\phi_{PSO}^{Fussed}$ feature vector. The ensembled feature vector $\phi^{Fused}$ is optimized using the PSO algorithm; this algorithm returned a feature vector with the dimensions N×428. The optimal set of features is utilized in this experiment for the evaluation of the PSO optimization algorithm performance. For the evaluation of PSO-optimized features, we have utilized multiple classifiers. Similar to other experiments, the performance is evaluated using the same performance measures such as the ACC, SEN, PRE, SPE, F1-Score, and execution time. The results depict that Q_SVM has achieved 100% accuracy in 64.238 s. E_S_Disc came in second, with an accuracy of 99.93%, and F_Tree achieved the third-highest accuracy of 99.92%. With an accuracy of 85.81%, C_Tree performed the worst in terms of classification. Table 4 displays the experiment’s findings.

**Figure 11:**
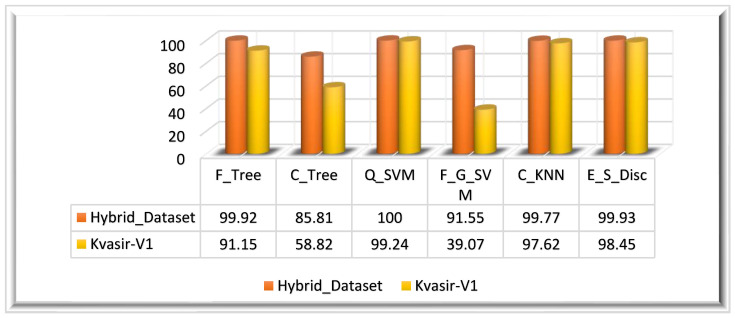
Experiment 4 results.

Experiment 3 is also evaluated on the Kvasir_V1 dataset. The PSO-optimized feature vector $\phi_{PSO}^{Fussed}$ is obtained by employing the PSO algorithm on the ensemble feature vector. The ensemble feature vector optimized using the PSO algorithm returned a feature vector of M×525 dimensions. The highest classification accuracy of this experiment (Kvasir_V1 dataset) is 99.24%, achieved on Q_SVM classifiers in 23.957 s. On the Kvasir_V1 dataset, the E_S_Disc classifier achieved the second-highest accuracy of 98.45% in 66.56 s, and C_KNN achieved the thirst-highest accuracy of 97.62%. F_G_SVM performed worst among all classifiers by achieving a 39.07% accuracy in 229.41 s. The visual representation of the results is given in Figure 11 for both datasets.

## 5. Discussion and Comparison with Existing Methods

The proposed approach is compared with the most recent methods in this section [1,3,19,33,35,42,43,56,57]. The authors in [3] proposed a method for gastrointestinal disease detection from WCE images, and they achieved a 98.40% accuracy. In [3], Khan et al. achieved a 98.40% accuracy. The methodology proposed in [35] is evaluated on the three datasets. This method achieved 99.25%, 99.90%, and 100.0% accuracies on the Kvasir-V1, Nerthus, and CUI WAH WCE datasets. In another research study [19], two datasets were utilized for the assessment of the proposed method; the considered datasets were Kvasir-V1 and CUIWAH WCE. The achieved accuracy on the CUI WAH WCE dataset was 99.80%, whereas on Kvasir-V1, an 87.80% accuracy was obtained. In [33], the authors achieved a 99.80% accuracy for the WCE image classification. Khan et al. [43] utilized the Hybrid dataset comprising 15,000 images and achieved a 99.50% accuracy. In [56,57], the authors proposed a method for classifying gastrointestinal disease detection and classification; they achieved 97% and 96.46% accuracies on the Kvasir-V1 dataset, respectively. Later on, the authors in [42] proposed a novel method for the GIT disease classification using two optimization algorithms, the proposed technique is named Moth-Crow-based features optimization. This approach outperformed the most recent methods on three datasets. This study used three datasets—CUI WAH WCE, Kvasir-V1, and Kvasir-V2—and achieved 99.42%, 97.85%, and 97.20% accuracies, respectively. In this research, we have utilized two datasets for the evaluation of the proposed method, which are the Hybrid Dataset (CUI WAH WCE, Kvasir-V1, CVC-Clinic) and the Kvasir-V1 dataset. Our contribution to this research is in the preprocessing phase, which returned a better set of features, which results in better performance. We have achieved 100% accuracy on the Hybrid Dataset and 99.24% accuracy on the Kvasir-V1 dataset in 58.140 and 23.957 s respectively.

Table 5 provides a comparison of the results obtained by the proposed method and existing methods. It is observed from the comparison that our proposed method has performed good on the Kvasir-V1 dataset in terms of all the performance measures, if we compare the performance with the results of state-of-the-art research studies [19,42,56,57]. One of our previous studies achieved a 99.25% accuracy, which is 0.01% better than that of the present research due to the complexity of this method. The proposed method achieved the results in less time as compared to the previous method. This is the limitation of the proposed method that can be addressed in a future research study by using the more advanced algorithms. In this research, we have utilized a hybrid dataset that comprises the three different dataset images. The proposed method has achieved 100% accuracy on the hybrid dataset in 58.140 s, which is a good achievement. It is evident from this comparison that our proposed approach has outperformed the most recent ones in terms of performance.

## 6. Conclusions

This research proposes a deep learning model of 23 layers named XcepNet23 for Gastrointestinal disease detection and classification. We have proposed a hybrid image preprocessing framework that is employed on the augmented dataset. The proposed hybrid contrast stretching-based image enhancement is employed on the dataset. We have extracted the two types of CNN features in this research work. The first CNN feature vector is obtained using the Global Average pool layer of the proposed XcepNet23 model. The second CNN feature vector is obtained from the original augmented dataset by utilizing the fine-tuned ResNet18 model. The texture variation of the region of interest is a big challenge in the accurate recognition of gastrointestinal diseases. In this research, we have extracted the texture features using the LBP method. The obtained feature vectors are ensembled to obtain the strong set of features that incorporate the two types of CNN and texture features. The fused feature vector is optimized using three different nature-inspired meta heuristic optimization algorithms (BDA, MFO, PSO). The experiments are conducted on two datasets: the Hybrid dataset and the Kvasir-V1 dataset. The evaluation of the method is performed on three different feature vectors: the original ensemble feature vector, BDA-optimized ensemble feature vector, MFO-optimized ensemble feature vector, and PSO-optimized ensemble feature vector. In light of the results of the comparison between the proposed method and the techniques that were already proposed, it is concluded that our method has performed better across all performance metrics. This comparison demonstrates that our proposed strategy has performed better than the most recent ones. In 58.140 and 23.957 s, respectively, we were able to achieve 100% accuracy on the Hybrid Dataset and 99.24% accuracy on the Kvasir-V1 dataset.

The only limitation of this research work is the segmentation of the gastrointestinal tract abnormalities. The most noticed abnormalities are bleeding, ulcers, and polyps. The segmentation of bleeding, ulcers, and polyps can be performed along with the recognition of diseases in future research work. It is difficult to detect three different types of diseases using the same methodology, as these three types of abnormalities have different color, texture, and shape variations. The algorithm can be expanded in future research to identify and categorize a wider variety of gastrointestinal disorders. This might entail gathering more labeled data for less prevalent diseases or researching transfer learning strategies to use information from similar disorders. To implement the approach in clinics and enable real-time prediction, methods can also be researched. Moreover, in future work, this method may be improved by reducing the computational complexity.

## Figures and Tables

**Table 1:** Results on the $\phi^{Fused}$ feature vector.

Dataset Name	Classifier	ACC (%)	SEN (%)	PRE (%)	SPE (%)	F1 (%)	Kappa Score	MCC	Time (s)
**Hybrid_Dataset**	**F_Tree**	99.96	99.96	99.96	99.99	99.96	1.000	1.000	32.840
	**C_Tree**	82.39	75.45	87.64	95.66	70.22	0.75	0.790	**21.435**
	**Q_SVM**	**100.0**	**100.0**	**100.0**	**100.0**	**100.0**	1.000	1.000	124.94
	**F_G_SVM**	91.26	89.04	95.34	97.54	91.25	1.000	1.000	1286.8
	**C_KNN**	99.76	99.76	99.74	99.94	99.75	1.000	1.000	786.61
	**E_S_Disc**	99.98	99.98	99.98	99.99	99.98	1.000	1.000	483.66
**Kvasir_V1**	**F_Tree**	91.50	91.49	91.57	98.78	91.52	1.000	1.000	20.989
	**C_Tree**	59.45	59.45	65.68	94.2	55.35	0.7142	0.7412	**17.428**
	**Q_SVM**	**98.60**	**98.60**	**98.60**	**99.80**	**98.60**	1.000	1.000	41.758
	**F_G_SVM**	37.17	37.17	76.83	91.02	37.32	0.000	0.000	232.78
	**C_KNN**	97.97	97.97	98.06	99.71	97.99	1.000	1.000	56.647
	**E_S_Disc**	98.47	98.47	98.47	99.78	98.47	1.000	1.000	173.25

**Table 2:** Results on the $\phi_{BDA}^{Fussed}$ feature vector.

Dataset Name	Classifier	ACC (%)	SEN (%)	PRE (%)	SPE (%)	F1 (%)	Kappa Score	MCC	Time (s)
**Hybrid_Dataset**	**F_Tree**	99.91	99.91	99.91	99.97	99.91	1.000	1.000	14.220
	**C_Tree**	85.73	78.75	90.95	96.45	73.94	0.750	0.791	**7.8833**
	**Q_SVM**	**100.0**	**100.0**	**100.0**	**100.0**	**100.0**	1.000	1.000	58.140
	**F_G_SVM**	90.96	88.67	95.22	97.46	90.93	1.000	1.000	506.73
	**C_KNN**	99.72	99.73	99.70	99.93	99.71	1.000	1.000	264.70
	**E_S_Disc**	99.97	99.97	99.97	99.99	99.97	1.000	1.000	114.66
**Kvasir_V1**	**F_Tree**	91.62	91.62	91.82	98.80	91.71	1.000	1.000	12.289
	**C_Tree**	59.72	59.72	77.70	94.24	47.16	0.571	0.604	**11.308**
	**Q_SVM**	**98.40**	**98.40**	**98.37**	**99.77**	**98.38**	1.000	1.000	22.685
	**F_G_SVM**	44.90	44.90	73.70	92.12	42.78	0.428	0.495	92.103
	**C_KNN**	97.87	97.89	97.97	99.69	97.92	1.000	1.000	28.063
	**E_S_Disc**	98.32	98.32	98.32	99.76	98.32	1.000	1.000	66.739

**Table 3:** Results on the $\phi_{MFO}^{Fussed}$ feature vector.

Dataset Name	Classifier	ACC (%)	SEN (%)	PRE (%)	SPE (%)	F1 (%)	Kappa Score	MCC	Time (s)
**Hybrid_Dataset**	**F_Tree**	99.96	99.96	99.96	99.99	99.96	1.000	1.000	11.559
	**C_Tree**	85.66	78.68	90.82	96.44	73.85	0.750	0.791	**11.000**
	**Q_SVM**	**100.0**	**100.0**	**100.0**	**100.0**	**100.0**	1.000	1.000	67.941
	**F_G_SVM**	91.56	89.31	95.46	97.63	91.45	1.000	1.000	563.31
	**C_KNN**	99.79	99.8	99.76	99.94	99.78	1.000	1.000	302.73
	**E_S_Disc**	99.97	99.97	99.97	99.99	99.97	1.000	1.000	135.34
**Kvasir_V1**	**F_Tree**	91.90	91.90	92.02	98.84	91.95	1.000	1.000	**13.461**
	**C_Tree**	59.20	59.20	74.32	94.17	50.62	0.571	0.625	5.6968
	**Q_SVM**	**98.37**	**98.37**	**98.35**	**99.76**	**98.36**	1.000	1.000	36.044
	**F_G_SVM**	36.22	36.22	77.41	90.88	36.23	0.000	0.000	102.24
	**C_KNN**	97.42	97.45	97.58	99.63	97.47	1.000	1.000	28.223
	**E_S_Disc**	**98.42**	**98.42**	**98.45**	**99.77**	**98.43**	1.000	1.000	60.429

**Table 4:** Results on the $\phi_{PSO}^{Fussed}$ feature vector.

Dataset Name	Classifier	ACC (%)	SEN (%)	PRE (%)	SPE (%)	F1 (%)	Kappa Score	MCC	Time (s)
**Hybrid_Dataset**	**F_Tree**	99.92	99.92	99.91	99.98	99.92	1.000	1.000	**13.431**
	**C_Tree**	85.81	78.87	91.13	96.47	73.98	0.750	0.791	9.9464
	**Q_SVM**	**100.0**	**100.0**	**100.0**	**100.0**	**100.0**	1.000	1.000	64.238
	**F_G_SVM**	91.55	89.26	95.46	97.62	91.4	1.000	1.000	560.76
	**C_KNN**	99.77	99.77	99.75	99.94	99.76	1.000	1.000	296.58
	**E_S_Disc**	99.93	99.93	99.93	99.98	99.93	1.000	1.000	132.16
**Kvasir_V1**	**F_Tree**	91.15	91.15	91.21	98.73	91.17	1.000	1.000	11.495
	**C_Tree**	58.82	58.82	64.93	94.11	53.31	0.704	0.7313	**7.3797**
	**Q_SVM**	**99.24**	**98.69**	**98.69**	**99.87**	**98.69**	1.000	1.000	23.957
	**F_G_SVM**	39.07	39.07	78.32	91.29	39	0.142	0.285	92.005
	**C_KNN**	97.62	97.62	97.71	99.66	97.65	1.000	1.000	25.571
	**E_S_Disc**	98.45	98.45	98.47	99.77	98.46	1.000	1.000	66.56

**Table 5:** Comparison with Existing Techniques.

Ref. No.	Year	Dataset	Total Images	Dataset Classes	ACC (%)	SEN (%)	PRE (%)	SPE (%)	F1-Score	Time (s)
[3]	2020	CUI WAH WCE	6000	3	98.40	98.33	98.36	-	98.34	-
[1]	2021	CUI WAH WCE	4500	3	99.54	100.0	99.51	-	-	31.700
[19]	2021	Kvasir-V1	4000	8	87.80	87.40	87.99	98.06	87.63	50.700
		CUI WAH WCE	2326	3	99.80	99.83	99.80	99.92	99.81	17.031
[33]	2021	CUI WAH WCE	4000	4	99.80	99.00	99.25	-	99.12	211.90
[35]	2021	Kvasir-V1	4000	8	99.25	99.30	99.89	99.25	-	24.958
		Nerthus	5524	4	99.90	99.75	99.84	99.94	-	35.033
		CUI WAH WCE	2326	3	100.0	100.0	100.0	100.0	-	7.5840
[43]	2021	Hybrid Dataset	15,000	5	99.50	99.50	96.00	-	97.70	48.830
[56]	2021	Kvasir-V1	4000	8	97.00	96.80	-	99.20	-	-
[57]	2022	Kvasir-V1	4000	8	96.46	94.46	94.46	-	94.46	-
[42]	2022	CUI WAH WCE	4000	8	99.42	-	-	-	-	91.013
		Kvasir-V1	4000	8	97.85	-	-	-	-	71.031
		Kvasir-V2	8000	8	97.20	-	-	-	-	111.13
[58]	2022	Kvasir-V1	4000	8	98.20	-	-	-	-	52.504
		Kvasir-V2	8000	8	98.02	-	-	-	-	102.502
		CUI WAH WCE	-	3	99.61	-	-	-	-	69.544
Proposed		Hybrid Dataset	15,448	5	100.0	100.0	100.0	100.0	100.0	58.140
		Kvasir-V1	4000	8	99.24	98.69	98.69	99.87	98.69	23.957

## Data Availability

Dataset utilized in this research is publically available.
